# Extrarenal Angiomyolipoma: differential diagnosis of retroperitoneal masses

**DOI:** 10.1590/S1677-5538.IBJU.2016.0670

**Published:** 2018

**Authors:** Marcelo Wroclawski, Willy Baccaglini, Cristiano Linck Pazeto, Cristina Carbajo, Chaline Matushita, Arie Carneiro, Alexandre Pompeo, Sidney Glina, Antonio Carlos Lima Pompeo, Lívia Barreira Cavalcante

**Affiliations:** 1 Hospital Israelita Albert Einstein, São Paulo, SP, Brasil; 2 Departamento de Urologia, Faculdade de Medicina do ABC, Santo André, SP, Brasil; 3 Centro de Imuno-Histoquímica, Citopatologia e Anatomia Patológica (CICAP) São Paulo, SP, Brasil

## INTRODUCTION

Angiomiolipomas (AML) are benign mesenchymal tumors of unknown origin, that consist of mature adipose tissue, muscle fibers and blood vessel with thickened wall (1).

Renal AMLs represent 1% of renal tumors. However, extra-renal AMLs are extremely rare, and 60 cases have been described. Most extra-renal AMLs were observed at liver (18 patients) and at retroperitoneum (16 cases) (2). We report a retroperitoneum extra-renal AML.

## CASE REPORT

A fifty-one years old man with right lumbar pain for one day was submitted to computer tomography that showed an incidental left retroperitoneal nodule, in close contact to ipsilateral adrenal gland.

**Figure 1:**
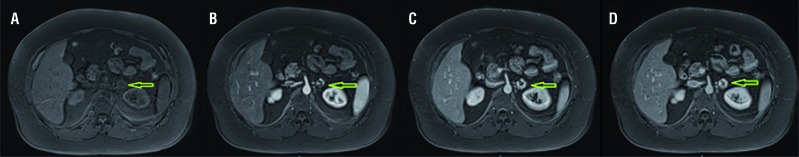
Magnetic ressonance with axial T1WI image in phases before contrast (A), arterial (B), Portal (C) and Equilibrium (D), showing a heterogenous nodule, hypervascularized, with peripheral progressive highlight due to contrast at the retroperitoneum, measuring 2.4x2.1cm.

**Figure 2:**
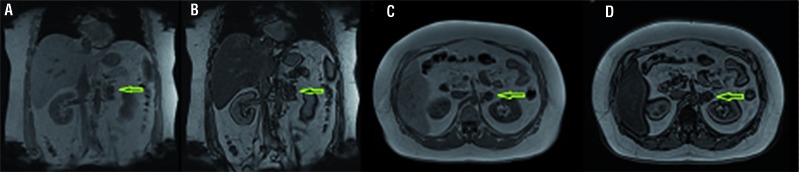
Magnetic resonance images (ECO Gradient sequences GRE) T1W1 inside and outside of phase (A and B) and in T2 with and without fat suppression (C and D); they show sparse areas of signal loss corresponding to lipomatous foci.

Magnetic resonance confirmed the presence of a heterogeneous nodule, close to left adrenal, hypervascularized, with sparse focus with loss of signal in sequences with fat saturation, with approximately 2.4x2.1cm (Figures 1 and 2). Blood analysis excluded a functional adrenal tumor.

**Figure 3:**
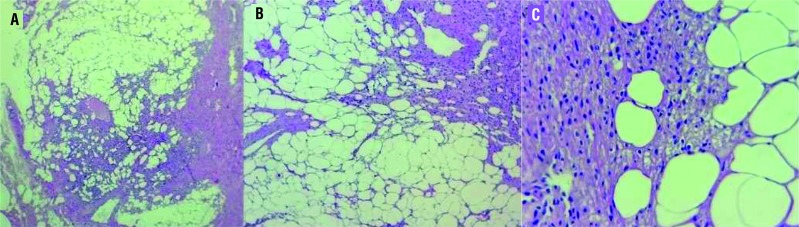
A) triphasic tumor, including mature adipose tissue, blood vessels with thickened wall and smooth muscle cells; B) component of elongated smooth muscle cells irradiating from gibbous vessels walls, permeating adipose tissue; c) angiomiolipoma, interface between mature fat cells and smooth muscle cells without atypia.

The lesion was excised by transperitoneal laparoscopy without complications, and it was diagnosed a mesenchymal lesion compatible to AML, confirmed by immune-histochemical assay (Table-1 and Figure-3).

**Table 1:** Immuno-histochemical results that confirm angiomiolipoma.

ANTIGEN	RESULT
HMB45	Reactivity in rare cells
Melan A	Negative
CD34	Positive
Smooth muscle actine	Positive

## DISCUSSION

AMLs are mainly asymptomatic incidentalomas. However, it was reported spontaneous bleeding (particularly in tumors with >4cm diameter), thromboembolic events and compression of adjacent structures (3–6). Also, the risk of malignization of such tumors is unknown.

Most reported retroperitoneal AMLs include symptomatic and big tumors (2); the present tumor was a small incidental lesion.

Image exams with presence of macroscopic fat are not conclusive, since liposarcoma represent most of retroperitoneal sarcomas adjacent to adrenal gland that can be confused to myelolipoma, particularly in well-defined lesions such as the one here described (7). Other possible diagnosis include lipomas, lymphoma, adenocarcinoma metastasis and germ cell tumors, extra-gonadal dermoid cyst, hibernomas and lipoblastomas, among others (8).

Percutaneous biopsy may be inconclusive; therefore, treatment of choice must be excision, preferably by minimally invasive technique. In the present case, lesion excision allowed histologic confirmation, preclude follow-up with image exams and had low morbidity to patient.

It is not uncommon the need of immune-histochemical exams for diagnosis, due to histologic similarities with other tumors, such as liposarcoma, leiomyoma and lipoma (9).
